# Clinical presentation, microbiological aetiology and disease course in patients with flu-like illness: a post hoc analysis of randomised controlled trial data

**DOI:** 10.3399/BJGP.2021.0344

**Published:** 2022-01-25

**Authors:** Theo J Verheij, Daniela Cianci, Alike W van der Velden, Christopher C Butler, Emily Bongard, Samuel Coenen, Annelies Colliers, Nick A Francis, Paul Little, Maciek Godycki-Cwirko, Carl Llor, Sławomir Chlabicz, Christos Lionis, Pär-Daniel Sundvall, Lars Bjerrum, An De Sutter, Rune Aabenhus, Nicolay Jonassen Harbin, Morten Lindbæk, Dominik Glinz, Heiner C Bucher, Bernadett Kovács, Bohumil Seifert, Ruta Radzeviciene Jurgute, Pia Touboul Lundgren, Muireann de Paor, Veerle Matheeussen, Herman Goossens, Margareta Ieven

**Affiliations:** Julius Center for Health Science and Primary Care, University Medical Center Utrecht, Utrecht, the Netherlands.; Julius Center for Health Science and Primary Care, University Medical Center Utrecht, Utrecht, the Netherlands.; Julius Center for Health Science and Primary Care, University Medical Center Utrecht, Utrecht, the Netherlands.; Nuffield Department of Primary Care, University of Oxford Medical Sciences Division, Oxford, UK.; Nuffield Department of Primary Care, University of Oxford Medical Sciences Division, Oxford, UK.; Department of Primary and Interdisciplinary Care (ELIZA) — Centre for General Practice, University of Antwerp Faculty of Medicine and Health Sciences, Antwerp, Belgium.; Department of Primary and Interdisciplinary Care (ELIZA) — Centre for General Practice, University of Antwerp Faculty of Medicine and Health Sciences, Antwerp, Belgium.; Primary Care and Population Sciences, University of Southampton, Southampton, UK.; Primary Care and Population Sciences, University of Southampton, Southampton, UK.; Division of Public Health, Centre for Family and Community Medicine, Medical University of Lodz, Lodz, Poland.; University of Copenhagen, Copenhagen, Denmark; University Institute in Primary Care Research Jordi Gol, Via Roma Health Centre, Barcelona, Spain.; Department of Family Medicine, Medical University of Bialystok, Bialystok, Poland.; Clinic of Social and Family Medicine, University of Crete School of Medicine, Heraklion, Greece.; Research and Development Primary Health Care, Västra Götalandsregionen, University of Gothenburg; Sahlgrenska Academy, Department of Public Health and Community Medicine/Primary Health Care, Institute of Medicine, Gothenburg, Sweden.; University of Copenhagen, Copenhagen, Denmark.; Department of Public Health and Primary Care, Ghent University Faculty of Medicine and Health Sciences, Ghent, Belgium.; University of Copenhagen, Copenhagen, Denmark.; Antibiotic Center for Primary Care, Department of General Practice, Faculty of Medicine, University of Oslo, Oslo, Norway.; Antibiotic Center for Primary Care, Department of General Practice, Faculty of Medicine, University of Oslo, Oslo, Norway.; Institute for Clinical Epidemiology and Biostatistics, University Hospital Basel, Basel, Switzerland.; Institute for Clinical Epidemiology and Biostatistics, University Hospital Basel, Basel, Switzerland.; Drug Research Center, Balatonfüred, Hungary.; Department of General Practice, Charles University, Prague, Czech Republic.; JSC Mano Seimos Gydytojas, Klaipeda, Lithuania.; Départment de Santé Publique, Université Côte d’Azur Faculté de Médecine, Nice, France;; Royal College of Surgeons in Ireland, Dublin, Ireland.; Laboratory of Medical Microbiology, Vaccine & Infectious Diseases Institute, University Hospital Antwerp, Antwerp, Belgium.; Laboratory of Medical Microbiology, Vaccine & Infectious Diseases Institute, University Hospital Antwerp, Antwerp, Belgium.; Laboratory of Medical Microbiology, Vaccine & Infectious Diseases Institute, University Hospital Antwerp, Antwerp, Belgium.

**Keywords:** diagnosis, oseltamivir, primary health care, randomised controlled trial, respiratory tract infections

## Abstract

**Background:**

There is little evidence about the relationship between aetiology, illness severity, and clinical course of respiratory tract infections (RTIs) in primary care. Understanding these associations would aid in the development of effective management strategies for these infections.

**Aim:**

To investigate whether clinical presentation and illness course differ between RTIs where a viral pathogen was detected and those where a potential bacterial pathogen was found.

**Design and setting:**

Post hoc analysis of data from a pragmatic randomised trial on the effects of oseltamivir in patients with flu-like illness in primary care (*n* = 3266) in 15 European countries.

**Method:**

Patient characteristics and their signs and symptoms of disease were registered at baseline. Nasopharyngeal (adults) or nasal and pharyngeal (children) swabs were taken for polymerase chain reaction analysis. Patients were followed up until 28 days after inclusion. Regression models and Kaplan–Meier curves were used to analyse the relationship between aetiology, clinical presentation at baseline, and course of disease including complications.

**Results:**

Except for a less prominent congested nose (odds ratio [OR] 0.55, 95% confidence interval [CI] = 0.35 to 0.86) and acute cough (OR 0.42, 95% CI = 0.27 to 0.65) in patients with flu-like illness in whom a possible bacterial pathogen was isolated, there were no clear clinical differences in presentations between those with a possible bacterial aetiology compared with those with a viral aetiology. Also, course of disease and complications were not related to aetiology.

**Conclusion:**

Given current available microbiological tests and antimicrobial treatments, and outside pandemics such as COVID-19, microbiological testing in primary care patients with flu-like illness seems to have limited value. A wait-and-see policy in most of these patients with flu-like illness seems the best option.

## INTRODUCTION

Evidence-based antibiotic use reduces an important driver of antimicrobial resistance and unnecessary exposure to side effects, and leads to better resource utilisation. In primary care unnecessary antibiotic use is common, especially for patients with respiratory tract infections (RTIs).^1^^,^^2^ It is commonly assumed that distinguishing viral from bacterial pathogens will lead to only those patients with a potential bacterial pathogen being considered for treatment, as those with a viral aetiology are unlikely to receive benefit from antibiotic therapy. The need for point-of-care tests to distinguish between bacterial and viral infections in primary care is therefore felt useful by many and the focus of several studies (https://www.value-dx.eu).^3^^,^^4^ However, there is a paucity of evidence about the relationship between aetiology, illness severity, and the clinical course of RTIs in primary care.

In a previous study (undertaken by the same authors) of lower RTIs in primary care, discoloured sputum was the only feature independently related to isolation of a probable bacterial pathogen, but this weak association had limited clinical utility.^5^ Furthermore, the illness course of adult primary care patients with an identified potential bacterial pathogen was compared with the illness course of those in whom no bacterial pathogen was detected and no difference was found in duration of symptoms, although those with a potential bacterial pathogen identified had slightly more severe symptoms at day 2 to 4.^6^ However, in that study the illness course in those with a viral aetiology was not compared with the illness course in those with a potential bacterial pathogen and those with a potentially dual (viral and bacterial) aetiology, and the study was limited to adults with lower RTIs. Thus, it is important to study the relationship between presentation and course of disease, and microbiological aetiology to support the development of relevant diagnostic and therapeutic strategies for common RTIs in primary care. Therefore, in the current study the clinical presentation and illness course in patients with flu-like illness in whom a viral, a bacterial, and a dual infection was identified are compared.

**Table:** How this fits in

Both GPs and patients still assume that a distinction between viral and bacterial infections is important for illness prognosis and treatment decisions. In this article, the presence of viral and bacterial pathogens was looked at in relation to illness severity and course of disease in patients with flu-like illness. The results show that there were no meaningful differences in illness severity at presentation and course of disease between patients in whom viral, bacterial, or mixed pathogens were found. Outside specific circumstances, such as the current COVID-19 pandemic, the distinction between viral and bacterial respiratory infections in patients with flu-like illness does not seem clinically relevant.

## METHOD

Data used in this analysis were collected during an open-label, pragmatic, adaptive, randomised controlled trial on the additional effects of oseltamivir to usual care (symptomatic treatment and/or wait and see in almost all participants) in patients aged ≥1 year and presenting with flu-like illness in primary care. Flu-like illness was defined as a sudden onset of self-reported fever, with at least one respiratory symptom (cough, sore throat, or running or congested nose) and one systemic symptom (headache, muscle ache, sweats or chills, or tiredness), with symptom duration of ≤72 hours during a seasonal flu epidemic at baseline. The primary endpoint of the trial was time to recovery, defined as return to usual activities, with fever, headache, and muscle ache minor or absent.^7^

Between 15 January 2016 and 12 April 2018, 3266 participants in 15 European countries were recruited during three seasonal flu seasons. The participants were allocated 1629 to usual care plus oseltamivir and 1637 to usual care; primary outcome in 1533 (94%) and 1526 (93%) was ascertained, respectively.^8^ A baseline case report form was completed covering overall clinician-rated severity of flu-like illness (GPs’ global impression of mild, moderate, or severe illness without provided, predefined criteria), duration of symptoms, comorbidity, temperature, pulse, individual symptom severity rating (patient-reported at inclusion), and usual care advice (registered by GP).

Oropharyngeal and nasal flocked swabs (COPAN, Brescia, Italy) were taken from participants aged <16 years, and flocked nasopharyngeal swabs (COPAN, Brescia, Italy) from those aged ≥16 years. Clinicians were trained in swabbing techniques using face-to-face and online video methods. The Fast Track Diagnostics Respiratory Pathogens 21 plus real-time polymerase chain reaction (PCR) assay (Fast Track Diagnostics, Luxembourg) was used for the qualitative detection of flu A, flu B, flu A H1N1, human coronaviruses NL63, 229E, OC43, and HKU1, paraflu viruses 1, 2, 3, and 4, human metapneumovirus A and B, rhinovirus, respiratory syncytial viruses A and B, adenovirus, enterovirus, parechovirus, bocavirus, *Mycoplasma pneumoniae*, *Chlamydia pneumoniae*, *Streptococcus pneumoniae*, *Haemophilus flue* B, and *S. aureus*, but results were not available for clinicians or for patients to influence management.

Patients were asked to complete a symptom diary for 14 days to indicate when they had returned to their usual daily activities and to evaluate fever, running or congested nose, sore throat, headache, cough, shortness of breath (adults only), muscle ache, sweats or chills (adults only), diarrhoea, nausea or vomiting, abdominal pain, low energy or tiredness, not sleeping well, dizziness, and feeling generally unwell. Symptoms were scored as either no, minor, moderate, or major problem. For children aged ≤12 years, the diaries were supplemented with child-specific questions from the Canadian Acute Respiratory Illness Flu Scale. Patients were contacted by telephone between days 2 and 4, days 14 and 28, and after 28 days to support study participation and diary completion, monitor intervention adherence, and ascertain a minimal outcome dataset.

### Data analysis

Baseline characteristics were summarised as patient counts and percentages. Symptom severity was dichotomised into major and moderate versus minor and no problem. A variable that indicates the viral aetiology (with or without a bacterial pathogen) was created as follows: ‘negative’ indicates that no viruses or bacteria were observed; ‘viral’ indicates that at least one virus was observed but no bacteria; ‘mixed’ indicates that in the sample at least one virus and at least one bacterium were present; ‘bacterial’ indicates no viruses were observed but at least one bacterium.

In order to investigate whether viral and/or a possible bacterial aetiology had a relationship with the severity of each symptom, logistic regression models were used. In the current study the term possible bacterial aetiology was used as it was recognised that in a minority of the patients bacterial carriership should be considered.

The investigated symptoms were fever, nasal congestion or runny nose, sore throat, cough, diarrhoea, headache, muscle aches and/or pains, low energy or tiredness, not sleeping well, and their severity (major and moderate versus minor and no problem). A model for each symptom was run and the variables included in the model were:

- the four combinations of presence and absence of viral and/or bacterial pathogen;
- age categorised as adults (≥12 years) and children (<12 years); and
- duration of flu-like illness symptoms at baseline (measured as 1, 2, or 3 days).

Results are expressed in terms of odds ratios (ORs), where 1 indicates that the viral/bacterial pathogen does not affect the outcome, OR >1 indicates that a specific viral/bacterial pathogen is associated with higher odds of the outcome, and OR <1 indicates that a specific viral/bacterial pathogen is associated with lower odds of the outcome. In this analysis, the focus was on differences between viral and possible bacterial infections, and the largest category ‘viral’ was chosen as the reference category.

The time to resumption of usual activities with fever, headache, and muscle ache being a minor or no problem was visualised with Kaplan–Meier curves for the four combinations of presence and absence of viral and/or bacterial pathogen. In addition, using the same outcome, a Cox model was generated including age group, treatment group (usual care plus oseltamivir and usual care only), the presence of comorbidities such as diabetes and chronic respiratory conditions, use of pain medications (defined as use of paracetamol, ibuprofen, or other pain medication, at least two doses in 1 day), or antibiotics and duration of flu-like illness symptoms (measured as 1, 2, or 3 days). Kaplan–Meier curves were also produced for the outcome resolution of minor or no problem for all of the following symptoms: fever, nasal congestion or runny nose, sore throat, headache, cough, muscle aches and/or pains, diarrhoea, low energy or tiredness, and not sleeping well.

It was also descriptively investigated whether clinically relevant complications were related to microbiology results. The statistical analyses were performed with SAS Enterprise Guide (version 8.2).

## RESULTS

There were 3266 participants who were included in the original trial described above. No pathogens, only a viral pathogen, only a bacterial pathogen, and both viral and bacterial pathogens were found in 849 (26.3%), 1949 (60.4%), 90 (2.8%), and 339 (10.5%) patients, respectively (Table 1). See also Supplementary Table S1 for an inventory of the different bacteria and viruses found. In total, therefore, 2288 (70.9%) patients had a viral pathogen detected and 429 (13.3%) had a bacterial pathogen detected. The majority of patients had a typical flu-like illness with fever, runny nose, and acute cough, together with fatigue and muscle ache.

**Table 1.** Baseline characteristics study participants (*N* = 3266)

**Characteristic^a^**	**Participants, *n* (%)**
**Age (years)**	
Child (>1 and ≤12)	479 (14.7)
Adult (>12)	2780 (85.3)

**Comorbidity**	
Diabetes	82 (2.5)
Chronic respiratory condition	196 (6.0)

**Virus/bacterium^b^**	
Negative	849 (26.3)
Viral	1949 (60.4)
Mixed	339 (10.5)
Bacterial	90 (2.8)

**Symptoms (major or moderate)**	
Fever	2551 (78.8)
Nasal congestion, runny nose	1991 (61.4)
Sore throat	1914 (59.5)
Headache	2379 (74.6)
Cough	2227 (68.7)
Muscle aches and/or pains	2286 (72.0)
Diarrhoea	170 (5.3)
Low energy, tiredness	2670 (82.6)
Not sleeping well	1733 (53.7)

a *Data missing.*

b *Negative: presence of no viruses and no bacteria. Viral: at least one virus and no bacteria. Mixed: at least one virus and at least one bacterium. Bacterial: no viruses and at least one bacterium.*

At baseline it was observed that, in patients in whom only bacterial pathogen were seen or no virus/bacteria were found, they had somewhat less severe nasal congestion and cough than those in whom only a viral aetiology was seen. Patients with a longer than average duration of symptoms before baseline had a more serious acute cough. No relevant difference was observed for the other symptoms. Irrespective of which pathogen was found, children and adults showed some differences at baseline, but without a clear consistent pattern (Table 2). In patients aged >75 years the results did not differ significantly from those in adults in other age groups (data not shown).

**Table 2.** Relationship between symptom severity at baseline and presence/absence of viruses and/or bacteria, age, and previous duration of flu-like illness symptoms^a^

**Symptoms (major or moderate), effect**	**Odds ratio**	**95% Wald confidence limit**	

		**Lower**	**Upper**
**Fever (*n*= 3199)**			
Negative versus viral	0.83	0.68	1.00
Bacterial versus viral	0.85	0.50	1.45
Mixed versus viral	1.33	0.93	1.91
Adult versus child^a^	0.66	0.48	0.89
Duration flu-like illness symptoms 2 versus 1	0.95	0.77	1.18
Duration flu-like illness symptoms 3 versus 1	0.85	0.68	1.05

**Nasal congestion, runny nose (*n* = 3204)**			
Negative versus viral^a^	0.56	0.48	0.66
Bacterial versus viral^a^	0.55	0.35	0.86
Mixed versus viral	1.02	0.77	1.35
Adult versus child^a^	0.71	0.56	0.90
Duration flu-like illness symptoms 2 versus 1	1.12	0.94	1.34
Duration flu-like illness symptoms 3 versus 1	1.16	0.96	1.39

**Sore throat (*n*= 3175)**			
Negative versus viral^a^	1.29	1.09	1.52
Bacterial versus viral	1.08	0.69	1.67
Mixed versus viral	0.96	0.73	1.26
Adult versus child	1.07	0.85	1.36
Duration flu-like illness symptoms 2 versus 1	0.95	0.80	1.14
Duration flu-like illness symptoms 3 versus 1	1.05	0.87	1.26

**Headache (*n*= 3152)**			
Negative versus viral	1.05	0.86	1.27
Bacterial versus viral	1.21	0.72	2.02
Mixed versus viral	1.01	0.75	1.36
Adult versus child^a^	2.07	1.61	2.66
Duration flu-like illness symptoms 2 versus 1	0.81	0.66	1.00
Duration flu-like illness symptoms 3 versus 1	0.81	0.66	1.00

**Cough (*n*= 3203)**			
Negative versus viral^a^	0.39	0.33	0.46
Bacterial versus viral^a^	0.42	0.27	0.65
Mixed versus viral	0.98	0.73	1.32
Adult versus child^a^	1.30	1.02	1.66
Duration flu-like illness symptoms 2 versus 1^a^	1.31	1.09	1.58
Duration flu-like illness symptoms 3 versus 1^a^	1.65	1.36	2.00

**Muscle aches and/or pains (*n*= 3140)**			
Negative versus viral	0.92	0.77	1.11
Bacterial versus viral	0.72	0.45	1.16
Mixed versus viral	0.84	0.63	1.12
Adult versus child^a^	3.71	2.90	4.75
Duration flu-like illness symptoms 2 versus 1	0.91	0.75	1.12
Duration flu-like illness symptoms 3 versus 1	0.85	0.69	1.04

**Diarrhoea (*n*= 3189)**			
Negative versus viral^a^	1.50	1.06	2.12
Bacterial versus viral	0.69	0.21	2.26
Mixed versus viral	0.94	0.51	1.74
Adult versus child	0.84	0.51	1.38
Duration flu-like illness symptoms 2 versus 1	1.30	0.85	1.98
Duration flu-like illness symptoms 3 versus 1^a^	1.62	1.07	2.45

**Low energy, tiredness (*n*= 3192)**			
Negative versus viral	1.02	0.82	1.27
Bacterial versus viral	1.38	0.77	2.49
Mixed versus viral	1.00	0.72	1.38
Adult versus child^a^	2.31	1.77	3.01
Duration flu-like illness symptoms 2 versus 1 day	1.18	0.94	1.49
Duration flu-like illness symptoms 3 versus 1 day	1.03	0.81	1.30

**Not sleeping well (*n*= 3188)**			
Negative versus viral	0.92	0.78	1.09
Bacterial versus viral	1.24	0.80	1.93
Mixed versus viral	1.14	0.87	1.48
Adult versus child^a^	1.34	1.07	1.69
Duration flu-like illness symptoms 2 versus 1 day	1.05	0.89	1.26
Duration flu-like illness symptoms 3 versus 1 day	1.16	0.97	1.38

a *Superscript ‘a’ denotes symptoms with a confidence interval that does not include 1.0. When the confidence interval of the odds ratio includes 1, the odds of having the symptom in both categories are similar. Negative = presence of no viruses and no bacteria. Bacterial = no viruses and at least one bacterium. Viral = at least one virus and no bacteria. Mixed = at least one virus and at least one bacterium.*

**Figure 1.**
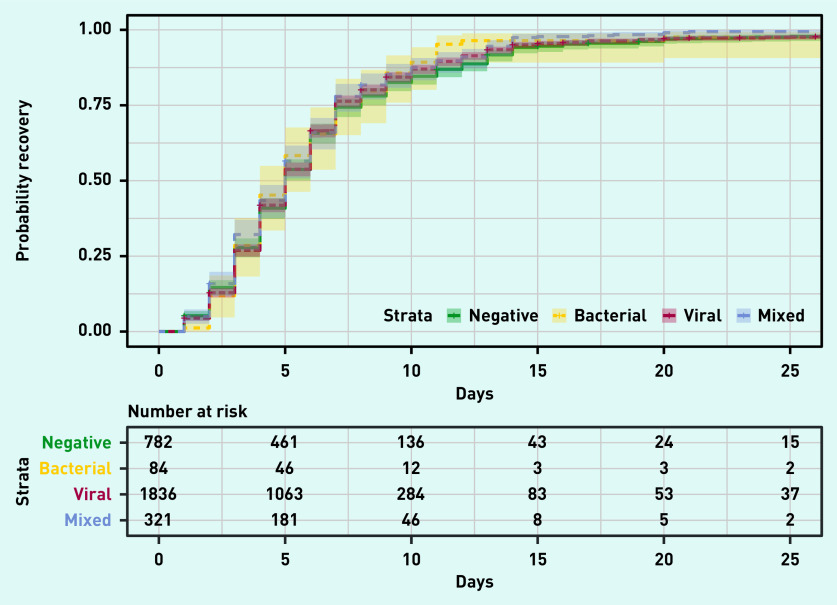
*Kaplan–Meier curve of time to recovery defined as return to usual activities, with fever, headache, and muscle ache minor or absent for each virus/bacterium class. The term ‘strata’ refers to the different levels that can be assumed by the variable that indicates the viral aetiology (with or without a bacterial pathogen), which are: viral: at least one virus and no bacteria; mixed: at least one virus and at least one bacterium; negative: presence of no viruses and no bacteria; bacterial: no viruses and at least one bacterium.*

Time to resume usual activities with fever, headache, and muscle ache being a minor or no problem did not differ significantly between patients, irrespective of whether a viral or bacterial pathogen or mixed infection was detected (Figure 1). These results did not change after adjusting for age, treatment group, comorbidities, medication taken, and duration of flu-like illness symptoms at baseline (see Supplementary Table S2). When resolution of all symptoms was examined, no differences between the different groups with presence/absence of viruses and/or bacteria were found (see Supplementary Figure S1).

Clinically relevant complications, such as the need for a hospital admission, were relatively few (64 patients) and did not seem to be related to microbiology results in study participants (data not shown).

## DISCUSSION

### Summary

Except for a somewhat less prominent congested nose and acute cough in patients with flu-like illness in whom a possible bacterial pathogen was isolated, there were no clear clinical differences in presentations between those with a possible bacterial aetiology compared with those with a viral one. Also, the course of disease and complications were not related to the aetiology identified by microbiology test results.

### Strengths and limitations

Strengths of this post hoc analysis are the sample size, the participation of a wide range of European countries, and real-time PCR tests of nasopharyngeal swabs taken in all adult patients (nasal and oropharyngeal swabs in children). Some limitations must also be taken into account when assessing the results of this study. First, study participants had flu-like illness, and very few of these had signs of pneumonia on clinical examination. Nevertheless, the results are generalisable to a large proportion of patients seen each winter season in primary care. Second, the study participants were included during flu seasons only, and therefore it could be that the proportion of viral infections could differ from respiratory infections outside flu seasons. However, in studies of community-acquired respiratory infections outside flu epidemics, viral infections are also far more common than bacterial infections.^9^^,^^10^ Third, some specific viral and bacterial infections could have a specific presentation and course of disease. Vos *et al* showed that common viruses other than flu account for a similar disease burden to flu infection.^11^ However, the current COVID-19 pandemic shows that new pathogens surely can have a specific morbidity and mortality, and testing for those new pathogens can of course be relevant. Fourth, both bacterial and viral strains that were identified could reflect asymptomatic carriage, and be unrelated to the signs and symptoms in the patient. This limitation is more important in children than in adults. In recent studies in adults, the asymptomatic carriage rates of *S. pneumococcus* (2.9%–5.6%), *H. influenzae* (1.4%), and viruses (4.3%) were lower than those found in the current study, suggesting that a substantial proportion represent true infections in the adult participants in the current study.^12^^,^^13^

### Comparison with existing literature

There are only a few studies that have been published on the relationship between microbiological test results in primary care patients and severity and course of disease. Vos *et al*, who compared the course of disease of lower RTIs of different viral aetiology, and Teepe *et al*, who studied the course of disease of bacterial lower RTIs, saw comparable survival curves as those found in the current study.^5^^,^^11^ Hopstaken *et al* also studied signs and symptoms in primary care patients with a lower RTI and could not find clinical predictors that could distinguish viral from bacterial infections, which is in line with the findings in the current study that viral and bacterial RTIs do not show relevant differences in clinical presentation.^14^ Voiriot *et al* studied patients with severe pneumonia admitted to an intensive care unit, and found that patients with a mixed viral/bacterial infection had more severe symptoms and a worse prognosis.^15^ In primary care patients with a much milder RTI this finding could not be confirmed.

### Implications for research and practice

The lack of relevant differences in severity at clinical presentation and course of disease between viral and bacterial infections in primary care patients with flu-like illness questions efforts to distinguish viral from potential bacterial pathogens. Identifying aetiology will only be useful if it has consequences for patient information or treatment. It has been found by this study group that oseltamivir can benefit older patients and those with comorbidity with flu-like illness. This effect was, however, not related to identified aetiology.^7^ Randomised controlled trials of antibiotic treatment for mild respiratory infections in primary care found no relevant benefit for patients with sinusitis, acute sore throat, or acute bronchitis.^16^^–^^18^ Studies exploring whether positive bacterial tests in mild respiratory infections modify the effects of antibiotic treatment found no or only modest effect modification. Seven studies have assessed the effects of antibiotics in patients with acute sore throat and positive throat swabs, and saw a somewhat milder and shorter course of disease, but irrespective of treatment 90% of patients were better by day 7.^18^ In patients with mild lower RTIs, Bruyndonckx *et al* found that there was a small beneficial effect of amoxicillin treatment in patients in whom a viral and a possible bacterial pathogen were detected but no beneficial effect of antibiotic treatment in all patients with a positive bacterial test.^19^ Meanwhile, it is obvious that in extraordinary situations such as the current COVID-19 pandemic, testing for specific pathogens, such as SARS-CoV-2, can be highly relevant for patient management and public health purposes. Recently Yu *et al* showed that budesonide had a beneficial effect in certain subgroups of patients with COVID-19.^20^

In conclusion, pathogen identification by laboratory PCR-based testing in primary care patients presenting with flu-like illness was not associated with meaningful differences in presentation or course of disease. Irrespective of aetiology, illness course was generally self-limiting and lasted for ≤14 days. A wait-and-see policy in most of these patients with flu-like illness seems the best option and, given the currently available antimicrobial treatments, and outside pandemics such as COVID-19, microbiological testing seems to have limited value.

## Data

After publication of the full trial report, formal requests for study data should be made to the corresponding author (Christopher C Butler) using a bespoke data request form delineating research aims, methods, and the variables needed. Such requests will be considered by the core ALIC^4^E team (Christopher C Butler, Theo J Verheij, Bohumil Seifert, Alike W van der Velden, and Emily Bongard) and the PREPARE coordinator (Herman Goossens). If research questions and methods are considered relevant and valid, the Data Management Department of the Julius Center, UMC Utrecht, will securely transfer the requested, fully anonymised data in the desired format to the party under data transfer agreements. The ALIC^4^E team will decide about co-authorships, after discussion with the interested party. The study protocol, statistical analysis plan, and informed consent form will be made available.
